# Insight into Patients’ Experiences of Cancer Care in Taiwan: An Instrument Translation and Cross-Cultural Adaptation Study

**DOI:** 10.3390/ijerph15081772

**Published:** 2018-08-17

**Authors:** Tsung-Hsien Yu, Kuo-Piao Chung, Yu-Chi Tung, Hsin-Yun Tsai

**Affiliations:** 1 Department of Health Care Management, National Taipei University of Nursing and Science, Taipei 108, Taiwan; ericthyu@ntu.edu.tw; 2 Institute of Health Policy and Management, College of Public Health, National Taiwan University, Taipei 100, Taiwan; yuchitung@ntu.edu.tw (Y.-C.T.); d98843003@ntu.edu.tw (H.-Y.T.); 3 Population Health Research Center, National Taiwan University, Taipei 100, Taiwan

**Keywords:** cancer patient experience, questionnaire development, survey

## Abstract

*Background*: Since Taiwan launched the Cancer Prevention Act in 2003, several prevention strategies and early detection programs have been implemented to reduce the incidence, morbidity and mortality rates of cancer. However, most of the programs have concentrated on healthcare providers. Evaluations from the patient’s perspective have been lacking. Thus, in this study a cancer patient experience questionnaire was developed in the Taiwanese context and a preliminary nationwide investigation was conducted on the status of cancer care from the patient’s perspective. *Methods*: An extensive literature review was first conducted to collect information on the existing instruments used to measure the cancer patient’s experience. Thereafter, a multidisciplinary expert panel was convened to select an optimal instrument based on the IOM’s six domains for evaluating patient-centered care. The European Organisation for Research and Treatment of Cancer (EORTC) translation procedure was applied to the questionnaire for cross-cultural adaptation. A nationwide field test was then implemented at certificated cancer care hospitals. *Results*: Fifteen questionnaires were collected for the literature review. The expert panel selected the National Cancer Patient Experience Survey based on the IOM’s recommendations. After cross-cultural translation of the questionnaire, a total of 4000 questionnaires were administered in 19 certificated cancer care hospitals and two major cancer patient associations, with 1010 being returned (25.25% response rate). Most of the respondents were middle-aged, and 70% were female. The respondents reported they had a good experience with cancer care, except for “Home care and support” and “Finding out what was wrong with you”. Stratified analysis was conducted, with the results showing that the cancer patients’ experiences varied depending on their sociodemographic and cancer-related characteristics. *Conclusions*: A Taiwanese version of the cancer patient experience survey questionnaire was developed. Its results showed that the cancer patient’s experiences varied, depending on the patient’s age, cancer type, and cancer history. This study can be used as a basis to establish a patient-centered care model for cancer care in Taiwan.

## 1. Introduction

Patient-centered care has become a focus of healthcare systems, since the publication of “Crossing the Quality Chasm”, a landmark report by the Institute of Medicine (IOM). The IOM defines patient-centered care as “care that is respectful of and responsive to individual patient preferences, needs, and values and ensures that patient values guide all clinical decisions” [1]. Many policies, programs, and activities have been implemented to improve patient engagement with care, such as shared decision-making, accountable care organization, and patient-centered medical homes. The benefits of implementing patient-centered care have gradually been revealed [2], especially in terms of patient satisfaction [3,4], self-care ability [5], and outcomes [5,6].

In the past, quality of care has been measured by the level of improvement to physical function and treatment outcomes, which have generally been easy for outsiders to measure and observe. Nonetheless, they have not captured the perceived quality of care from the patient’s perspective. The only way to measure this is to ask the patient and measure the patient-reported outcome. This approach has become mainstream, especially in cancer research [7,8] and many questionnaires have been developed, such as those focused on the patient experience [9,10,11,12,13,14,15,16,17,18,19,20,21,22] and quality of life [23,24,25]. Some studies have supported the idea that better survivorship comes along with better patient-reported outcomes, and patient-reported outcomes are routinely collected to improve the quality of care in some countries and regions, such as the U.S. (e.g., HCAHPS), Europe (Picker Survey of Patient Experiences), and the Netherlands (e.g., Quality of Care through Patient’s Eyes Program). However, these questionnaires, were developed in the West and thus require translation and cultural adaptation for use outside the Western setting.

Cancer has been the leading cause of death in Taiwan since the 1980s. According to the 2013 cancer registry annual report published by the Health Promotion Administration, there were nearly 100,000 newly diagnosed cancer patients in 2013, which was 1.5 times more than 10 years before. The mortality rate has been increasing too.

Since the launch of the Cancer Prevention Act in 2003, several prevention strategies and early detection programs have been implemented to reduce cancer incidence, morbidity and mortality rates, such as the cancer registry (Taiwan Cancer Database) and cancer care certification. Most efforts, however, have concentrated on healthcare providers and have not adequately addressed the patient’s perspective. The purpose of this study is to develop a cancer patient experience survey instrument for the Taiwanese context and conduct a preliminary nationwide investigation on the status of cancer care from the patient’s perspective.

## 2. Methods

The investigation involved two phases.

### 2.1. Phase 1: Instrument Development

#### 2.1.1. Candidate Survey Instrument Selection

The research team conducted a literature review through the Web of Science indexing service, well-known cancer patient-related organizations, and Google web search to identify the existing instruments measuring cancer patients’ experiences. A nine-member panel, comprising four patient association representatives, two cancer specialists, and three university professors, was convened to select the best questionnaire. A meeting was held, during which the IOM’s six domains for evaluating patient-centered care were introduced to facilitate discussion and help form a consensus [26]. The domains included: (1) respect for patients’ values, preferences, and expressed needs, (2) coordinated and integrated care, (3) provision of information, communication, and education, (4) physical comfort, (5) emotional support, and (6) involvement of family and friends.

#### 2.1.2. Survey Instrument Translation

Once the panel had selected the most optimal questionnaire, the research team contacted its owner for permission to use it. The EORTC Quality of Life Group translation procedure was then used to translate the questionnaire [27]. In the first step, two independent bilingual translators translated the questionnaire from English to Chinese. The two translators discussed any differences that were detected and if an agreement could not be reached, a third independent translator was invited to arbitrate. Thereafter, two other translators, both native English speakers with a high level of fluency in Chinese, translated the first translation from Chinese to English. The two translators discussed any differences in the translations and a third independent translator was invited to arbitrate if an agreement could not be reached. Finally, the research team convened a meeting of experts to review and finalize the survey questionnaire. The questionnaire was also pilot-tested to ensure that the questions were culturally appropriate for Taiwan.

### 2.2. Phase 2: Field Test and Preliminary Investigation

#### 2.2.1. Investigation Administration

Since 2008, when the cancer care certification system was initiated, 22 hospitals have been certified. All of the certificated hospitals were invited to participate in this study, and nineteen of them accepted. Two major nationwide cancer patient associations were also invited, to increase the number of participants. The investigation took place from March to June 2016. Within the hospitals, patients were identified as candidates if they had been outpatients during the time the questionnaires were being administered. Outpatient nurses delivered invitation letters to the cancer patients. If they agreed to participate, their informed consent was obtained and the survey questionnaire was given to them by an outpatient nurse. The patients were asked to take the questionnaire home and answer questions relating to their first experience with cancer treatment. Once the questionnaire was completed, the patient-respondents returned it to the research team’s office directly to avoid any unnecessary pressure or bias. Within the cancer patient association, the invitation letter, informed consent and survey questionnaire were delivered by the office staff. Otherwise, the procedure was the same as it was in the hospitals. The survey administration protocol was offered to both the nursing staff and office staff for consistency.

#### 2.2.2. Ethical Statement

The National Taiwan University Hospital Institution Review Board approved this study (protocol # 201501053RINA).

#### 2.2.3. Statistical Analysis

Statistical analyses were performed using SAS (version 9.4, SAS Institute Inc., Cary, NC, USA). In statistical testing, a two-sided *p*-value ≤0.05 was considered statistically significant. The distributional properties of the continuous variables were expressed as mean values and standard deviations (SD) and the categorical variables were presented as frequencies and percentages. An ANOVA test was carried out to examine the differences in the patients’ experiences, depending on their demographic and cancer-specific characteristics. A Scheffe’s test for post hoc comparison was also administered.

#### 2.2.4. Availability of data and materials 

All data generated during this study are included in this published article. The data and questionnaire analyzed during the current study are available from the corresponding author on reasonable request.

## 3. Results

### 3.1. Phase 1: Questionnaire Selection and Translation

A total of fifteen cancer patient’s experience questionnaires were collected during the search process. The consensus meeting was held on 4 December 2015. During the meeting, the research team presented the content of each candidate’s instrument, comparing the similarities and differences (see Table 1). Our panel agreed that the survey instrument should cover as many IOM domains as possible, but that the domains covered should vary (range: 2–6). “Provision of information, communication, and education”, “Respect for patients’ values, preferences, and expressed needs”, “Coordinated and integrated care”, and “Emotional support” were the most common domains. We also found that only Ouwens et al. [16] and the National Cancer Patient Experience Survey (NCPES) (version 2014) [28] covered all of the dimensions. Following a half-day discussion, the panel adopted the U.K.’s NCPES as the survey instrument for this study.

To evaluate the National Health Service’s (NHS’s) cancer services and its patients’ experiences during treatment, since 2010 Quality Health on behalf of the U.K.’s Department of Health, has administered the NCPES annually from September to December. The NCPES consists of the following 16 categories (80 items): seeing your GP (four items), diagnostic tests (five items), finding out what was wrong with you (five items), deciding the best treatment for you (six items), clinical nurse specialist (four items), support for people with cancer (seven items), operations (four items), hospital doctors (five items), ward nurse (four items), hospital care and treatment (10 items), home care and support (two items), hospital care as a day patient/outpatient (four items), outpatient appointments with doctors (two items), care from your general practice (two items), and your overall NHS care (six items). Each item contained three to six options, ranging from very positive to very negative experience. The questionnaire was designed to monitor national progress on cancer care and provide information on how to improve the quality of care. The results have been used for public reporting and have been provided to all NHS trusts. This questionnaire has also been used in Australia, New Zealand, Qatar, and other countries.

#### Questionnaire Translation

First, the research team contacted the instrument’s owner for authorization to use it. Next, The EORTC Quality of Life Group translation procedure was adopted to ensure that the questionnaire items were culturally appropriate for Taiwan. The questionnaire owner was also invited three times to engage in this phase via video conferencing. Eight members of our panel (four patients, two cancer specialists, and two university professors) convened to review and finalize the Taiwanese version of the cancer patient’s experience questionnaire.

On the recommendation of the experts, some of the items were removed due to differences in culture (e.g., what name you prefer to be called, sexual orientation, etc.) or health care systems (e.g., questions related to waiting time). We also added some questions on cancer, such as type, stage, and history, in the category of “about you”. In the end, 16 categories and 82 items comprised the Cancer Patient Experience Survey Taiwan version. The comparison between the two countries is presented in Table 2.

Before the investigation, 16 patients introduced by the cancer patient association were invited to pre-test the questionnaire. Their suggestions were used to modify the wording of some questions. As the Cancer Patient Experience Survey is a survey instrument, rather than a scale (i.e., it is used to collect information about the cancer treatment journey, rather than attitudes or beliefs), we only examined the Cronbach’s α. The value of Cronbach’s α in this instrument was 0.80, in line with the U.K. version (Cronbach’s α = 0.83).

### 3.2. Phase 2: Field Test and Preliminary Investigation

Nineteen hospitals (seven medical centers, 11 regional hospitals, and one community hospital) agreed to participate in the study. The questionnaire was administered from March to June 2016 through outpatient nursing staff, except as otherwise noted. Four thousand questionnaires were administered and 1010 were returned, a response rate of 25.25%.

Table 3 presents the results of the descriptive analysis. Around 60% of our respondents were middle-aged, 70% were female, and 90% lived with family. Thirty percent of the respondents had full time or part time jobs. Homemakers and retired people respectively accounted for 25%, and 18% were unemployed, the major reason being health issues. Forty percent of the respondents reported long-standing conditions. As for cancer specific characteristics, 84% of the respondents were being treated for cancer for the first time. The majority of cancer types were breast (51.5%) and colorectal (20.4%). Around 54% of the respondents were diagnosed at stages 1–2, and 30% were diagnosed at stages 3–4. The cancer history of our respondents was evenly distributed. In term of their experience with cancer care, the category “Before confirmed diagnosis” presented the distribution of each item, because both items asked for facts, rather than experiences. In this category, 40% of the respondents forgot how many times they had visited a doctor for health problems caused by cancer before they received a confirmed diagnosis, however, almost 60% saw a doctor within three months of when they felt something was wrong. The remaining categories showed the percentage of positive experience because they were composed of items related to the experience of cancer care. 

The results showed that the range of positive experiences among these categories was wide. The top three were “Outpatient appointments with doctor (82.4%)”, “Hospital care and treatment (81.8%)”, and “Hospital doctor (80.9%)”. The percentage of positive experience for “Home care and support” and “Finding out what was wrong with you” were below 60%; they were 47.3% and 57.4% respectively.

Table 4 shows the results of the sociodemographic-stratified analysis. We found that people in the senior group generally had more positive experiences than others, and in some categories females had more positive experiences than males. Living with family or not appeared to be unassociated with positive experience, except for the category “Support for people”. In terms of employment status, 10 out of 14 categories were unassociated with the cancer experience, and the associations between the remaining categories and cancer experience were inconsistent. As for long-standing conditions, nine out of 14 categories were unassociated with the cancer experience. The remaining categories showed that the respondents with long-standing conditions had a fewer positive experiences than the respondents without long-standing conditions, except for the category “Outpatient appointment with doctor”.

Table 5 shows the results of the cancer specific-stratified analysis. The experiences in some categories varied among cancer type, and there was often no difference in the experiences of first time cancer patients, non-first time cancer patients and those at different stages of cancer. Finally, in eight out of 14 categories, shorter cancer history was positively associated with a more positive experiences related to cancer care, except for the category “Outpatient appointment with doctor”.

## 4. Discussion

Patient-centered care is perhaps one of the most important goals for healthcare system reform in this century. To achieve this goal, the entire healthcare system must be redesigned. Cancer is a critical and complex illness and cancer patients would benefit from a holistic care model, such as patient-centered care. A more holistic care model would improve patient-reported outcomes, such as quality of life [29], survivorship [29], self-management [30], and other outcomes of care [5]. In this study, we developed a cancer patient’s experience questionnaire for Taiwan and conducted a large-scale field test. To the best of our knowledge, this questionnaire is the first cancer patient’s experience questionnaire adapted for use in the Eastern context. Although generalizability might be a major challenge, the results of the field test offer insight into contemporary cancer care from the patient’s perspective. The results of stratified analysis can also help different stakeholders, such as hospital staff or those working in health administration, develop strategies to improve patients’ perceptions of cancer care. In summary, our respondents reported they had a good experience with cancer care, except for the categories “Home care and support” and “Finding out what was wrong with you”. Our findings also showed that the cancer patients’ experiences varied depending on their sociodemographic and cancer-related characteristics. Respondents with different ages, cancer types, and cancer histories had different perceptions in roughly half of the categories.

Most of the existing studies adopting the NCPES as their data source have only explored the relationship between the patient’s characteristics (i.e., ethnicity [31,32,33,34], gender [32,33,34], age [32,33], and disability [32]) and a specific item in the questionnaire. In addition, there has been no study in which the relationship between cancer-specific characteristics and experience with cancer care have been discussed. Therefore, it is not easy for this study to compare its results with others, even the U.K. NCPES, which only calculated the percentage of positive experiences for each item, and reported the results item by item. Nonetheless, we may still compare our results to studies from relevant fields, to explain our findings.

As it is in many advanced countries, cancer is one of the major causes of death in Taiwan. Following the 2003 launch of the Cancer Prevention Act in Taiwan, many strategies were introduced to enhance the quality of care and cancer prevention, including encouraging cancer screening, cancer hospital certification, and a referral network for cancer care. Cancer hospital certification has had a particularly important effect on improving the quality of cancer care. Given that the sample in this study was collected from certified hospitals, there may have been a better quality of cancer care. This finding is similar to previous studies [35,36] and aligns with our finding that patients who have been diagnosed more recently have had a better experience.

There are, however, some issues worth raising. First, in terms of the questionnaire selection, we chose an existing questionnaire for the survey instrument. Although this approach is quicker and cheaper than creating a new instrument [37], cultural adaption is critical when using an instrument in a different context [38,39]. In this study, we focused on questionnaire translation and involved different stakeholders in selecting the questionnaire. Four bilingual translators assisted with forward and back translations and the questionnaire owner was invited to join in the translation process to ensure the translation did not deviate significantly from the NCPES questionnaire. A pilot test was also conducted with 16 cancer patients. Thus, the validation of the survey instrument was appropriate.

The second issue is the response rate. The NCEPS in the U.K. has a 60–70% response rate. Compared to this, our response rate appeared to be inadequate. The major reason for the difference is that the NCPES in the U.K. is implemented by the U.K. Department of Health as part of its national policy. Our study was just a research project, so we could not ask all patients to take part in the survey. In addition, the low response rate might have been due to two reasons. The first was the authentic intention to take the survey. The patients who agreed to participate in this study might have done so out of courtesy, or were too shy to reject the invitation on the spot. Plus, it was a take home survey (the same as in the U.K.). Therefore, patients who were unlikely to join this survey, might not have completed it. Second, we did not have the contact information of the patients who answered our survey questionnaire, so we could not contact the participant patients who did not mail their questionnaire back to our office.

The third issue concerns the definition of “positive experience” in each category. Most questions in the NCPES had four or five options, with the U.K. Department of Health reporting the proportion of patients who provided responses indicating “good” or “excellent” (the definition of positive experience) for each item [40]. That is also the way we did it in this study. However, how does one elaborate the patient’s overall experience for each dimension or their journey with cancer care? As mentioned above, most of the exiting studies, even the U.K. NCPES, did not calculate the percentage of positive experience for each category. Too many survey items might make interpretation difficult. Thus, we adopted an equal weighting approach, which is one of most commonly used composite scores, to calculate the overall experience of each dimension. As a result, this study was not only the first experience survey for cancer care from the eastern world, but also the first study to present the data in a holistic manner.

### Limitations

Although a rigorous approach was followed to develop a cancer patient’s experience survey in the Taiwanese context, some limitations should be noted. First, we relied extensively on stakeholders and experts to select and translate the survey instrument. Undoubtedly, different experts might have produced a different outcome. To address this challenge, we sought to invite a broad range of stakeholders and experts. Second, for reasons of feasibility and convenience, we conducted a purposive sampling at the hospital level. The selection bias and low response rate might limit the generalizability of the study. Third, recall bias might also exist because the respondents were asked to provide detailed information on cancer screening. Some respondents, especially those with a longer cancer history, might have had trouble recalling this information. Recall bias, however, might have been unavoidable. Fourth, the socio-demographic information was insufficient. Both the U.K. and Taiwan version of the NCPES faced this challenge. Due to the length of the survey, some interesting information was not collected, such as educational and income levels. This limited the scope of analysis, and should be taken into account in future surveys. Finally, the questionnaire was completed by a relative or friend, which was inevitable.

## 5. Conclusions

Understanding the patient experience is not only important to evaluating the quality of care from the patient’s perspective, but is also necessary to achieve patient-centered care. This study provides insight into cancer care in Taiwan from the patient’s perspective. Further, the results may provide a basis for establishing a patient-centered care model for cancer care in Taiwan. We suggest that the health authority should pay more attention to the categories in which most respondents did not perceive a positive experience, for example, providing an incentive for hospitals and the community to work together after patients are discharged. In addition, healthcare providers are encouraged to review whether their care process meets the needs of patients with varied characteristics.

## Figures and Tables

**Table 1:** Comparison of existing questionnaires.

Questionnaire/Source	Dimension	Number of Items	IOM’s Six Recommendations					
			Respect for Patients’ Values, References and Expressed Needs	Coordinated and Integrated Care	Provision of Information, Communication and Education	Physical Comfort	Emotional Support	Involvement of Family and Friends
Perceived Physician’s Communication Style Scale [19]	4	27	V		V		V	
OPPQNCS [17]	4	40	V	V	V		V	V
M-PICS [18]	4	20	V		V			
REPERES-60 [14]	4	60	V	V	V		V	
CQI-BC [12]	15	118	V	V	V		V	
PCQ-P [20]	5	106	V	V	V	V		V
MCQ [15]	3	15	V	V			V	
QUOTE ^chemo^ [21]	7	67	V		V	V	V	V
QUOTE Breast Cancer [13]	5	33	V	V	V			
Pain CQ [10]	2	33	V	V	V	V		V
Ouwens et al. [16]	7	56	V	V	V	V	V	V
Young et al. [22]	2	20		V	V		V	V
APECC [9]	6	33	V	V	V		V	
SAT-RAR [11]	4	23			V	V	V	
NCPES [28]	16	80	V	V	V	V	V	V

**Table 2:** Comparison between U.K. and Taiwan versions of Cancer Patient Experience Survey.

Category	Number of Items in U.K. Version	Number of Items in Taiwan Version
1. Seeing your GP	4	2
2. Diagnostic tests.	5	5
3. Finding out what was wrong with you.	5	5
4. Deciding the best treatment for you.	6	7
5. Clinical nurse specialist.	4	4
6. Support for people with cancer.	7	7
7. Operations.	4	4
8. Hospital doctors.	5	5
9. Ward nurse.	4	4
10. Hospital care & treatment.	10	9
11. Home care and support.	2	2
12. Hospital care as a day patient/outpatient.	4	4
13. Outpatient appointments with doctor.	2	2
14. Care from your general practice.	2	2
15. Your overall NHS/ NHI care.	6	6
16. About you	10	13

**Table 3:** Descriptive analysis.

**Numbers of respondent**	**1010**
**Socio-demographic characteristics**	
Age, %(n)	
<40	12.0 (121)
40–60	59.5 (601)
>60	22.1 (223)
Unknown	6.4 (65)
Gender (Female), %(n)	70.0 (706)
Live with family (yes), %(n)	91.7 (926)
Employment status, %(n)	
Full-time	23.2 (234)
Part-time	7.2 (73)
Homemaker	23.9 (241)
Retired	22.5 (227)
Unemployed—and seeking work	2.3 (23)
Unemployed—unable to work for health reasons	16.0 (162)
Others	2.5 (25)
Unknown	2.5 (25)
Long-standing conditions (yes), %(n)	41.7 (421)
**Cancer-specific characteristics**	
First time been treated for cancer(yes), %(n)	84.1 (849)
Cancer type, %(n)	
Breast cancer	520 (51.5)
Colorectal cancer	206 (20.4)
Others	284 (28.1)
Cancer stage, %(n)	
0	6.7 (68)
1–2	53.7 (542)
3–4	29.7 (300)
Unknown	9.9 (100)
Cancer history, %(n)	
<1 year	33.8 (341)
1–5 years	35.3 (356)
>5 years	29.3 (296)
Unknown	1.7 (17)
**Cancer experience**	
Before confirmed diagnosis	
Number of times to see doctor about health problem caused by cancer before confirmed diagnosis, %(n)	
1–2 times	33.5 (338)
>2 times	25.0 (252)
Unknown	41.6 (420)
Duration between when first thought something wrong and first visit to a hospital doctor, %(n)	
<3 months	56.9 (575)
>3 months	31.7 (320)
Unknown	11.4 (115)
Positive experience of Diagnostic tests, mean (S.D.)	67.3 (37.0)
Positive experience of Finding out what was wrong with you, mean (S.D.)	57.4 (38.3)
Mean % of Positive experience of Deciding the best treatment for you, mean (S.D.)	64.3 (33.6)
Positive experience of Case manager, mean (S.D.)	73.1 (39.8)
Positive experience of Support for people with cancer, mean (S.D.)	76.0 (28.2)
Positive experience of Operations, mean (S.D.)	74.7 (35.7)
Positive experience of Hospital doctors, mean (S.D.)	80.9 (28.0)
Positive experience of Ward nurse, mean (S.D.)	73.3 (31.2)
Positive experience of Hospital care and treatment, mean (S.D.)	81.8 (20.9)
Positive experience of Home care and support, mean (S.D.)	47.3 (44.2)
Positive experience of Hospital care as a day patient/outpatient, mean (S.D.)	66.2 (40.2)
Positive experience of Outpatient appointments with doctor, mean (S.D.)	82.4 (36.3)
Positive experience of Care from your general practice, mean (S.D.)	74.6 (39.9)
Positive experience of Overall cancer care experience, mean (S.D.)	65.3 (32.7)

**Table 4:** Experience of cancer care: categories vs. cancer-specific characteristics.

Category	Age				Gender			Live with Family			Employment Status				Long-Standing Condition		
	G1:	G2:	G3:		G1:	G2:		G1:	G2:		G1:	G2:	G3:		G1:	G2:	
	<40	40–60	60+	sig ^§^	Female	Male	sig ^¶^	Yes	No	sig ^¶^	Em ^1^	H/R ^2^	Unem ^3^	sig ^§^	Yes	No	sig ^¶^
Diagnostic tests, mean (S.D.)	60.1 (37.0)	66.4 (37.2)	71.4 (36.5)		70.0 (36.6)	66.2 (37.1)		67.4 (36.9)	65.6 (37.9)		69.3 (36.5)	66.8 (36.9)	65.2 (37.6)		65.9 (37.7)	68.4 (36.4)	
Finding out what was wrong with you, mean (S.D.)	49.5 (38.5)	55.2 (38.7)	64.1 (36.1)	** G3 > G2 G3 > G1	62.0 (37.4)	55.2 (38.5)	*	57.2 (38.5)	56.3 (35.1)		58.5 (37.8)	55.8 (38.0)	58.1 (39.5)		55.6 (37.9)	58.3 (38.6)	
Deciding the best treatment for you, mean (S.D.)	60.2 (34.6)	62.1 (33.7)	69.6 (32.7)	** G3 > G2 G3 > G1	67.8 (32.1)	62.6 (34.2)		64.3 (33.5)	61.1 (34.9)		66.5 (33.5)	62.9 (33.8)	63.6 (32.9)		61.9 (33.9)	65.7 (33.4)	
Clinical nurse specialist, mean (S.D.)	65.0 (44.8)	71.0 (40.5)	79.9 (35.4)	** G3 > G2 G3 > G1	80.2 (34.8)	70.1 (41.4)	***	73.5 (39.8)	69.1 (39.3)		76.5 (38.5)	70.7 (41.2)	74.1 (39.0)		73.6 (39.4)	72.8 (40.0)	
Support for people, mean (S.D.)	73.8 (28.4)	76.9 (28.2)	74.9 (28.2)		75.1 (28.6)	76.5 (28.0)		76.6 (27.8)	69.6 (32.0)	*	75.9 (27.0)	75.5 (29.2)	78.6 (27.2)		74.4 (28.8)	77.4 (27.6)	
Operations, mean (S.D.)	71.0 (40.0)	73.6 (35.6)	76.3 (35.7)		74.1 (37.6)	75.0 (34.8)		75.1 (35.6)	69.4 (37.6)		75.1 (35.3)	75.5 (34.4)	72.8 (38.9)		72.1 (37.3)	76.4 (34.6)	
Hospital doctors, mean (S.D.)	75.9 (30.2)	79.6 (28.3)	85.9 (25.4)	** G3 > G2 G3 > G1	79.8 (29.9)	81.3 (27.1)		80.9 (27.8)	79.6 (31.7)		79.2 (29.8)	82.9 (25.4)	78.6 (30.1)		77.7 (31.0)	83.0 (25.6)	*
Ward nurse, mean (S.D.)	66.6 (30.1)	71.8 (31.6)	79.5 (29.9)	** G3 > G2 G3 > G1	74.4 (32.9)	73.0 (30.2)		73.3 (31.4)	75.3 (29.0)		74.5 (30.9)	74.5 (30.5)	69.3 (33.2)		69.9 (32.9)	75.9 (29.7)	*
Hospital care & treatment, mean (S.D.)	78.5 (20.1)	81.3 (21.8)	83.9 (19.3)		81.7 (20.9)	81.9 (21.0)		82.1 (20.6)	78.0 (24.5)		83.3 (19.5)	83.5 (19.4)	77.4 (24.7)	** G2 > G3 G1 > G3	80.2 (21.7)	83.0 (20.3)	
Home care and support, mean (S.D.)	35.9 (42.6)	45.1 (44.7)	55.2 (42.5)	** G3 > G2 G3 > G1	54.8 (43.5)	43.0 (44.0)	**	47.2 (44.0)	48.0 (46.3)		45.1 (45.2)	49.8 (43.6)	46.3 (44.6)		43.5 (42.9)	49.9 (44.8)	
Hospital care as a day patient/outpatient, mean (S.D.)	55.9 (42.7)	65.5 (39.5)	70.7 (40.7)	** G3 > G1	70.2 (39.7)	64.3 (40.4)	*	66.6 (39.9)	59.2 (44.1)		67.6 (37.9)	65.8 (41.0)	65.1 (41.3)		61.4 (41.4)	69.5 (39.1)	**
Outpatient appointments with doctor, mean (S.D.)	83.3 (35.7)	83.0 (36.1)	80.6 (38.0)		85.1 (34.0)	81.2 (37.4)		82.4 (36.5)	80.8 (35.0)		81.4 (37.3)	79.7 (38.4)	89.2 (29.3)	** G3 > G2	85.3 (34.0)	80.1 (38.0)	*
Care from your general practice, mean (S.D.)	72.3 (41.5)	73.0 (40.5)	79.6 (36.7)		77.2 (39.9)	72.8 (40.1)		74.9 (39.4)	66.7 (44.2)		64.1 (45.8)	79.0 (36.2)	76.9 (38.7)	** G2 > G1	72.4 (40.6)	75.9 (39.6)	
Your overall NHI care, mean (S.D.)	59.2 (35.7)	62.9 (33.1)	73.2 (29.7)	*** G3 > G2 G3 > G1	70.6 (32.8)	62.9 (32.4)	**	65.8 (32.6)	57.1 (32.9)	*	62.8 (33.9)	68.2 (31.2)	62.5 (33.0)	*	61.7 (33.7)	67.7 (31.8)	**

* *p* < 0.05 **; *p* < 0.01; *** *p* < 0.001; ^1^ Full time and part-time; ^2^ Homemaker and retired; ^3^ All unemployed and other; sig: significant ^§^ ANOVA **^¶^** *t*-test.

**Table 5:** Experience of cancer care: categories vs. cancer-specific characteristics.

Category	Cancer Type				First Treatment			Cancer Stage				Cancer History			
	G1:	G2:	G3:		G1:	G2:		G1:	G2:	G3:		G1:	G2:	G3:	
	BC	CRC	Other	sig ^§^	Yes	No	sig ^¶^	0	1–2	3–4	sig ^§^	<1	1–5	>5	sig ^§^
Diagnosis tests, mean (S.D.)	64.6 (37.7)	73.5 (33.4)	67.9 (37.5)		66.7 (36.9)	69.4 (37.8)		73.6 (36.1)	64.9 (37.1)	69.5 (36.8)		67.2 (37.1)	67.9 (36.7)	65.8 (37.4)	
Finding out what was wrong with you, mean (S.D.)	52.7 (38.6)	64.8 (35.7)	60.4 (38.5)	** G2 > G1	57.3 (38.3)	56.0 (38.3)		69.9 (35.7)	54.7 (38.1)	58.6 (38.5)	** G1 > G2	60.6 (37.1)	59.1 (37.6)	51.0 (39.7)	** G1 > G3 G2 > G3
Deciding the best treatment for you, mean (S.D.)	61.0 (34.1)	68.9 (32.3)	65.8 (32.8)	** G2 > G1	64.4 (33.4)	60.7 (34.5)		72.3 (30.9)	62.4 (33.6)	64.6 (33.7)		68.0 (31.6)	67.8 (31.9)	54.7 (35.6)	*** G1 > G3 G2 > G3
Clinical nurse specialist, mean (S.D.)	65.2 (43.5)	83.4 (31.4)	80.0 (34.9)	*** G2 > G1 G3 > G1	73.8 (39.3)	66.5 (43.4)		73.4 (42.5)	72.2 (40.0)	73.6 (39.4)		82.5 (32.3)	76.5 (36.8)	56.7 (46.7)	*** G1 > G3 G2 > G3
Support for people, mean (S.D.)	78.1 (26.6)	77.6 (27.7)	73.1 (30.2)	*** G2 > G3 G1 > G3	77.6 (27.1)	72.0 (31.2)	*	72.1 (30.4)	77.5 (27.4)	76.5 (27.8)		76.0 (28.6)	79.1 (29.3)	74.9 (28.6)	
Operations, mean (S.D.)	73.4 (36.2)	79.6 (32.4)	73.0 (38.1)		75.2 (35.2)	73.9 (38.8)		75.4 (36.7)	75.7 (34.7)	73.7 (37.4)		76.8 (34.2)	78.8 (33.7)	66.0 (39.7)	** G1 > G3 G2 > G3
Hospital doctors, mean (S.D.)	81.0 (26.5)	83.8 (26.8)	79.4 (30.1)		81.7 (27.4)	78.7 (29.2)		87.5 (23.2)	81.5 (26.8)	79.8 (29.7)		83.9 (25.3)	78.4 (30.9)	81.1 (25.9)	* G1 > G2
Ward nurse, mean (S.D.)	72.7 (29.2)	77.2 (31.4)	71.8 (32.0)		74.2 (30.4)	70.6 (31.8)		73.8 (32.7)	74.3 (29.2)	72.5 (32.5)		74.7 (30.3)	74.0 (30.8)	70.8 (31.1)	
Hospital care & treatment, mean (S.D.)	82.4 (20.3)	84.8 (19.6)	80.5 (20.4)	* G2 > G3	82.8 (20.0)	80.5 (21.4)		87.6 (14.8)	82.3 (19.9)	81.7 (21.5)		84.5 (19.2)	81.3 (20.5)	80.5 (21.4)	
Home care and support, mean (S.D.)	42.5 (44.3)	52.1 (44.2)	48.9 (43.6)		47.5 (44.4)	44.1 (43.2)		58.3 (45.4)	45.3 (44.7)	47.4 (43.1)		49.6 (45.1)	46.8 (43.4)	42.0 (43.7)	
Hospital care as a day patient/outpatient, mean (S.D.)	63.5 (40.0)	70.2 (39.8)	69.5 (40.2)		66.9 (39.7)	62.5 (42.3)		73.0 (39.4)	64.8 (40.4)	67.5 (39.5)		70.4 (39.0)	68.3 (39.4)	59.7 (41.2)	** G1 > G3
Outpatient appointments with doctor, mean (S.D.)	84.2 (34.4)	83.5 (35.6)	78.1 (40.5)		81.9 (36.8)	86.7 (32.9)		82.4 (34.3)	80.5 (38.3)	86.4 (32.7)		74.7 (42.1)	90.3 (28.6)	81.6 (36.0)	*** G2 > G1 G2 > G3
Care from your general practice, mean (S.D.)	71.1 (40.4)	75.5 (40.9)	75.7 (39.8)		72.9 (40.8)	78.8 (37.4)		75.0 (40.6)	74.6 (38.8)	72.1 (42.9)		69.7 (42.5)	76.0 (38.9)	75.9 (39.2)	
Your overall NHI care, mean (S.D.)	59.6 (32.5)	71.6 (32.1)	71.8 (31.0)	*** G2 > G1 G3 > G1	65.0 (32.8)	65.4 (31.6)		68.1 (32.3)	64.1 (32.4)	66.1 (33.1)		69.8 (30.9)	66.8 (32.6)	58.0 (33.3)	*** G1 > G3 G2 > G3

* *p* < 0.05; ** *p* < 0.01; *** *p* < 0.001; sig: significant ^§^ ANOVA ^¶^ *t*-test; NHI: Taiwan National Health Insurance.
